# Effects of scale of movement, detection probability, and true population density on common methods of estimating population density

**DOI:** 10.1038/s41598-017-09746-5

**Published:** 2017-08-25

**Authors:** David A. Keiter, Amy J. Davis, Olin E. Rhodes, Fred L. Cunningham, John C. Kilgo, Kim M. Pepin, James C. Beasley

**Affiliations:** 1 University of Georgia, Savannah River Ecology Laboratory, D.B. Warnell School of Forestry and Natural Resources, PO Drawer E, Aiken, SC 29802 USA; 2 United States Department of Agriculture, Animal and Plant Health Inspection Service, Wildlife Services, National Wildlife Research Center, 4101 Laporte Avenue, Fort Collins, CO 80521 USA; 3 University of Georgia, Savannah River Ecology Laboratory, Odum School of Ecology, PO Drawer E, Aiken, SC 29802 USA; 4 United States Department of Agriculture, Animal and Plant Health Inspection Service, Wildlife Services, National Wildlife Research Center, Mississippi Field Station, PO Box 6099, Mississippi State, MS 39762 USA; 5 United States Department of Agriculture, Forest Service, Southern Research Station, PO Box 700, New Ellenton, SC 29809 USA

## Abstract

Knowledge of population density is necessary for effective management and conservation of wildlife, yet rarely are estimators compared in their robustness to effects of ecological and observational processes, which can greatly influence accuracy and precision of density estimates. In this study, we simulate biological and observational processes using empirical data to assess effects of animal scale of movement, true population density, and probability of detection on common density estimators. We also apply common data collection and analytical techniques in the field and evaluate their ability to estimate density of a globally widespread species. We find that animal scale of movement had the greatest impact on accuracy of estimators, although all estimators suffered reduced performance when detection probability was low, and we provide recommendations as to when each field and analytical technique is most appropriately employed. The large influence of scale of movement on estimator accuracy emphasizes the importance of effective post-hoc calculation of area sampled or use of methods that implicitly account for spatial variation. In particular, scale of movement impacted estimators substantially, such that area covered and spacing of detectors (e.g. cameras, traps, etc.) must reflect movement characteristics of the focal species to reduce bias in estimates of movement and thus density.

## Introduction

Knowledge of population density is essential to the field of wildlife ecology, providing a foundation for effective planning of management and conservation and for basic ecological research. As such, numerous density estimators have been developed for broad and species- or situation-specific use^1, 2^. Because every density estimator operates according to a set of assumptions (e.g. capture-mark-recapture [CMR] estimators generally assume that marks are not lost or overlooked), which may be comparatively robust or weak, these estimators are likely to differ in their susceptibility to the effects of 1) ecological processes, such as animal movement, 2) observational processes, such as baseline detection rates, 3) ecosystem characteristics, such as underlying population density, and 4) the interactions of these factors. For this reason, evaluation of potential impacts of these processes on accuracy and precision of estimators is necessary.

Density, or abundance per unit area, is often the parameter of interest in wildlife studies, as it allows comparison among studies which might not have sampled the same size area and provides spatial context to resulting information^3, 4^. However, there are many challenges inherent to estimating density of wildlife populations. A well-established issue is variability in the observational process resulting in a detection probability (*p*) of <1.0; thus, jointly estimating detection with density is necessary^5^. One of the simplest estimators to account for imperfect detection of animals is the 2-sample Lincoln-Petersen estimator (LPE), which uses a ratio of marked to unmarked animals to estimate $\hat{p}$ and abundance^6^. More complex CMR estimators that explicitly model covariates affecting $\hat{p}$ and thereby refine estimates of density or abundance have since been developed^5, 7^, as have non-CMR methods^8^. Many of these techniques, however, do not explicitly account for movement of animals in estimating animal abundance. Conversion of resulting estimates of abundance to density, an inherently spatial metric, requires some knowledge of the scale of animal movement to determine the area to which inference about populations can be applied. The size of an animal’s home range relates to the scale at which an animal moves (i.e. a larger home range denotes that an animal has larger scale movements than one with a smaller home range). The general scale of movement for a population may greatly affect abundance estimates through changes in the availability of an animal to be sampled (i.e. an animal that is only present on a sampling grid for a short duration of the sampling period may not be detected as readily or frequently as one that is present on the sampling grid at all times^9^). This lack of geographic closure caused by animal movement is often addressed through ad-hoc estimation of the effective area sampled by a particular data collection method.

One common technique to determine the effective area sampled by abundance estimators is to buffer the convex polygon of the sampling grid by the mean maximum distance moved (MMDM) or half mean maximum distance moved (HMMDM) by animals during the study period^10, 11^. In contrast to post-hoc calculation of effective area sampled, spatially explicit capture-recapture (SECR) models allow for direct inference on effective sample area by accounting for spatial variability in the detection process, potentially resulting in improved inference about density of wildlife populations over traditional capture-recapture approaches^4^. Recent research has compared spatially explicit density estimators with non-spatial estimators (i.e. those that use an ad-hoc approach to estimate the effective area sampled)^12–19^, but few studies have evaluated the accuracy and precision of tested estimators (but see refs 13, 18, 19). Further, while studies frequently compare analytical techniques in their ability to estimate population density without bias, a greater understanding of the effects of the scale of animal movement, an underlying mechanistic ecological process, on estimator performance is necessary to guide estimator choice. In addition to animal scale of movement, population and environmental characteristics can affect overall detection rates, thereby influencing accuracy and precision of density estimators. Evaluation of both the effects of these processes and their interactions with animal movement on accuracy and precision of common density estimators and field-based comparison of density estimation techniques will allow researchers and managers to choose the most appropriate and applicable density estimator for their research conditions.

Wildlife population estimation methods have evolved over the last several decades to meet the challenges inherent in monitoring natural systems. Data collection for these methods may be noninvasive, in which capture and handling of animals is not required, potentially minimizing disturbance to populations^20^; or invasive, in which animals are captured. Common noninvasive methods include use of camera traps^16^, fecal pellet counts^21^, and collection of hair or scat for genetic testing^20^. Analytical techniques such as mark-resight^22^ and genetic CMR^23^ have been developed to estimate density from noninvasive data. Examples of invasive data gathering techniques include live-trapping and marking for analysis in a CMR framework^7^, use of biomarkers and a recapture event^24^ for analysis by LPEs^24^, and lethal removal or harvest of animals for analysis by removal models^25^. Each data collection technique has unique advantages and disadvantages that may make it more or less susceptible to effects of ecological processes and underlying ecosystem characteristics, yet it is rare that multiple combinations of field and analytical techniques are compared in their ability to estimate density^26, 27^.

In this study, we had two objectives: 1) to evaluate the robustness of a suite of common density estimators to changes in the scale of animal movement, underlying population density, probability of detection, and the interactions between these processes, and 2) to provide recommendations as to when application of each estimator is appropriate based upon simulation results and the observed practicality and feasibility of field implementation of each. We accomplish these objectives by employing common invasive and noninvasive field techniques and a suite of analytical techniques to estimate population density of a globally widespread species *Sus scrofa*, the wild pig, at three study sites (Table 1), and using the gathered data to parameterize simulations for evaluation of estimator robustness to changes in ecological and observational processes.

**Table 1:** Data sources, implementation, and references for tested density estimators, Savannah River Site, South Carolina, USA, 2015.

Analytical Technique	Field Data Used	Implementation	Citation
Biomarker LPE	Biomarker data, camera data, corral trap data	Simple function written in R^40^ or Excel	6, 7
Camera LPE	Camera data, corral trap data	Simple function written in R or Excel	6, 7
Camera SECR	Camera data	Package *secr* in R	42, 50
Trap SECR	Camera data, corral trap data	Package *secr* in R	42, 50
Removal	Corral trap data	Hierarchical Bayesian model using custom MCMC code written in R	8

## Results

### Density Estimates

**Figure 1:**
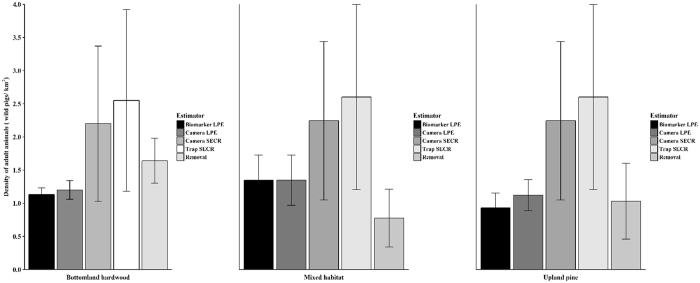
Estimated densities of wild pigs (*Sus scrofa*) at three study sites at the Savannah River Site using five analytical techniques, South Carolina, USA, 2015. Error bars represent 95% confidence intervals.

Density estimates from the five analytical techniques ranged from 0.91–2.60 adult animals/km^2^ in the three study sites tested (Fig. 1). Camera SECR and trap SECR models generally produced higher estimates of density than other methods. Estimates across study sites were similar within a given method (Fig. 1). Numbers of animals marked or captured by each field method in the three study sites are presented in the Supplementary Information (Appendix S1). In calculating false-positive and false negative rates of mark determination for the biomarker LPE, we found uncertainty in both the capture and recapture occasion. We, therefore, present a sensitivity analysis of the potential effects of this uncertainty on resulting density estimates in Appendix S2. Movement rates used to calculate buffer sizes for abundance estimators varied among habitat types (range of MMDM: 326–896 m), meaning the effective area sampled differed between study sites. Additional discussion of results specific to each field technique can be found in Appendix S1.

### Simulation

**Figure 2:**
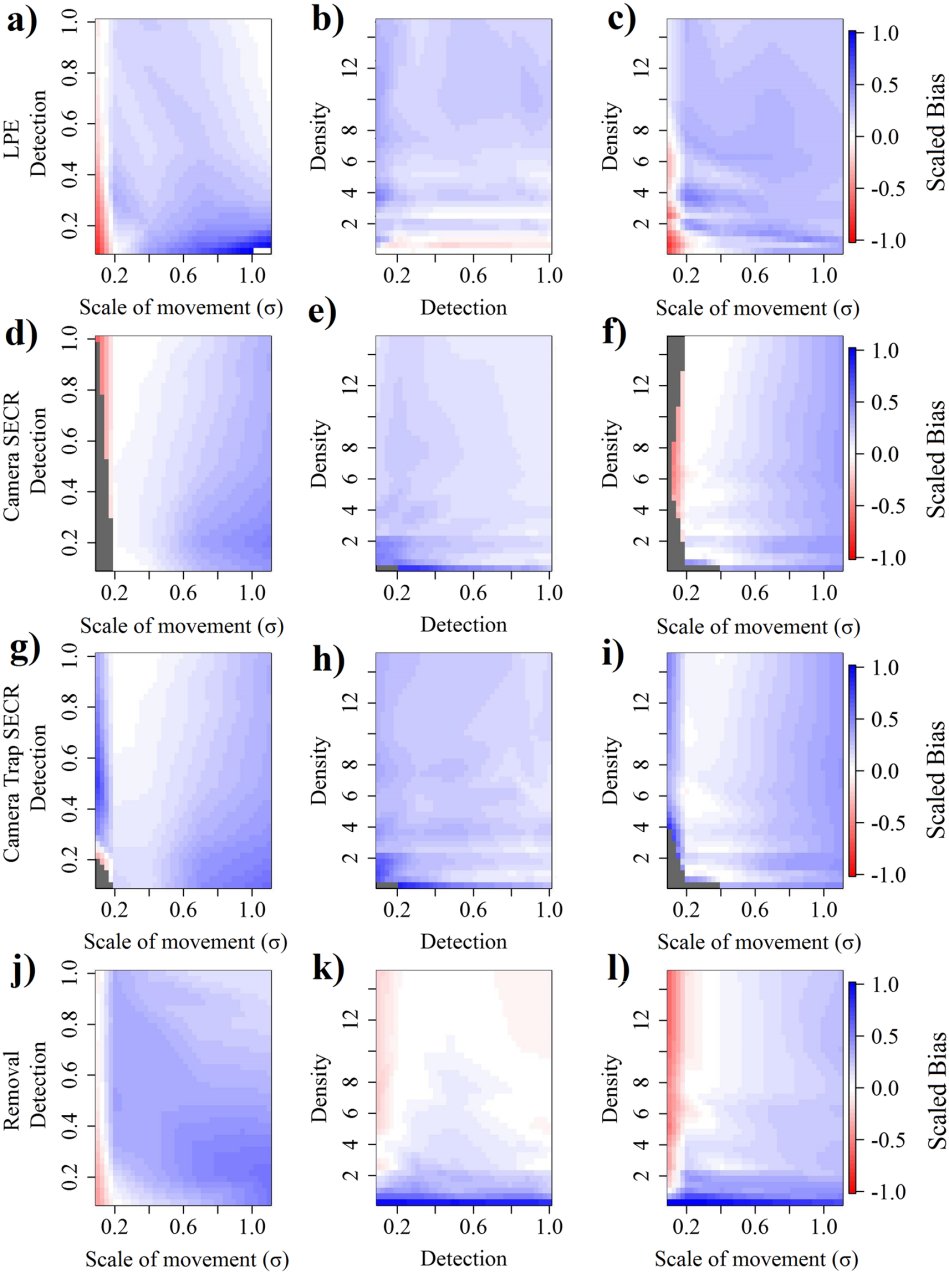
Effects of scale of movement, probability of detection, and density on scaled bias ((estimated density − true density)/true density) of tested analytical techniques from simulations. Parameter values at which models did not run are displayed in gray.

Simulations showed scale of movement often influenced the scaled bias of estimators more than probability of detection or true density for all estimators (Fig. 2). We found that LPEs were biased high when the scale of movement was high (Fig. 2a,c), biased low when scale of movement was low (Fig. 2a,c), and performed more poorly at low detection probabilities (Fig. 2a,b). Camera SECR and trap SECR models exhibited similar patterns to each other (Fig. 2d–i). Under some simulated conditions SECR models were not able to estimate density based on the sparseness of encounters (Fig. 2d–i), although trap SECR models, which employed greater amounts of data, were less affected (Fig. 2g,i). Camera SECR and trap SECR models tended to be biased high at high scales of movement and were unable to produce results when scales of movement were low (Fig. 2d–i). The removal estimator was more affected by the density parameter than the scale of movement or detection parameters, as shown by greater changes in the scaled bias as a result of change in density rather than the other parameters (Fig. 2k,l); removal models were biased high at low densities and biased low when scale of movement was low (Fig. 2j–l). Buffer choice to estimate effective area sampled had a substantial impact when converting abundance estimates to density. In particular for removal models when a naïve buffer was employed models performed poorly, but when an appropriate buffer based upon prior information (e.g. MMDM) was employed, the bias caused by animal movement was minimized (Appendix S1). We found MMDM buffers performed better than other buffer choices (i.e. HMMDM, naïve) in converting estimates of abundance to density (Appendix S1).

**Figure 3:**
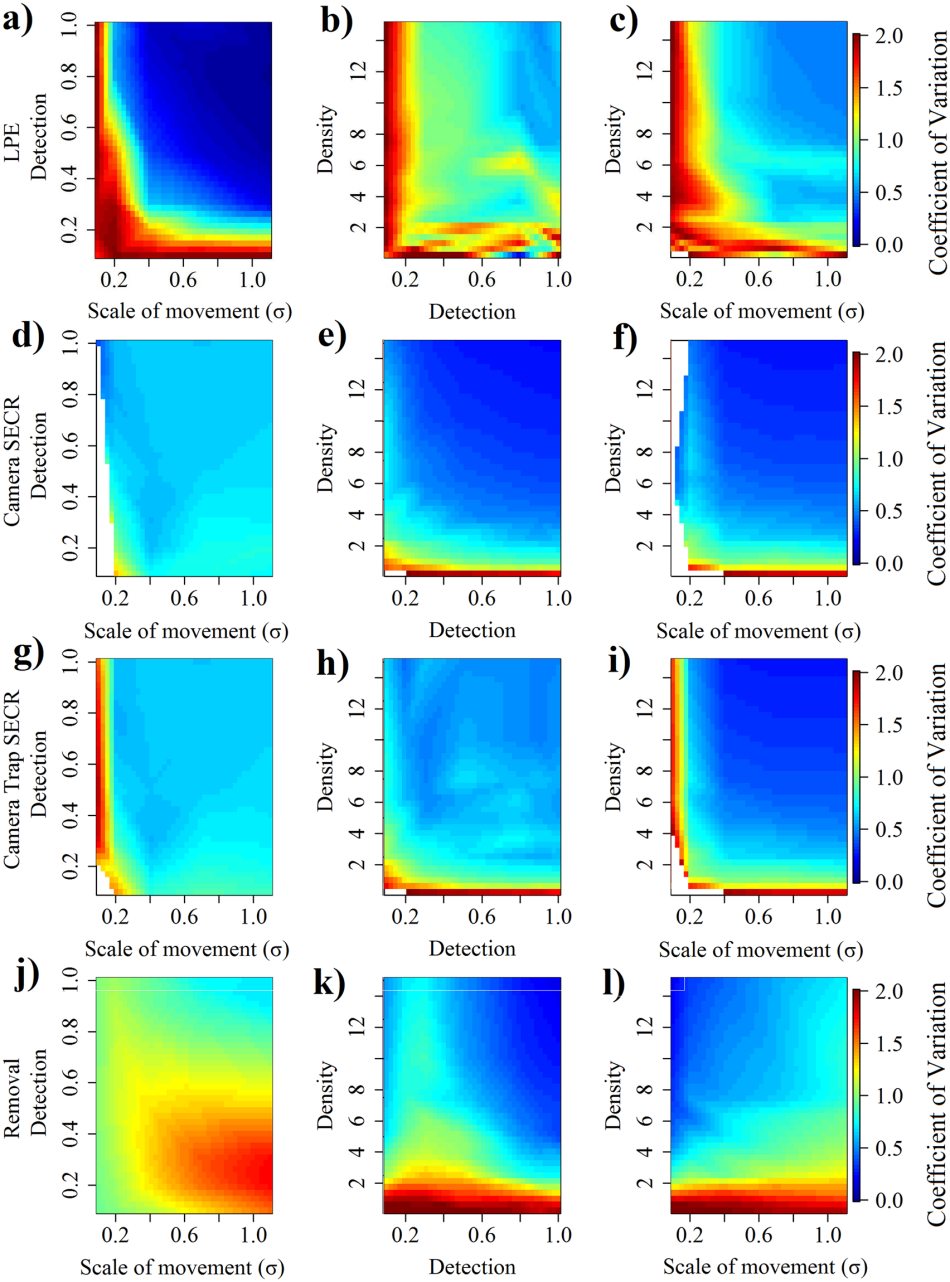
Effects of scale of movement, probability of detection, and density on the coefficients of variation (CV) of density of tested analytical techniques from simulations. The upper bound of CV values represents any values ≥2.0. Parameter values at which models did not run are displayed in gray.

In terms of precision, LPEs exhibited poor performance at low detection probabilities and low scales of movement (Fig. 3a–c). Further, when true variation was accounted for, inconsistent patterns in precision of LPEs emerged (Appendix S1). When data were sufficient to allow parameter estimation, camera SECR and trap SECR models were fairly consistent in estimating precision, and were most imprecise at low scales of movement and low abundances (Fig. 3d–i). The removal model was most imprecise at low densities and exhibited increased imprecision with increased scales of movement when detection rates were low (Fig. 3j–l; Appendix S1).

## Discussion

We employed and compared five methods of density estimation under field conditions and used simulations of ecological (e.g., scale of animal movement) and observational processes (e.g., baseline probability of detection) in lieu of known abundances to compare population density estimators under known conditions and evaluate their accuracy and relative strengths. Comparison of field methods to estimate animal density provides essential information for managers planning conservation or management programs and yet is infrequently conducted. Further, assessment of the accuracy of metrics is often impossible to perform in field conditions (but see refs 13, 18, 19), leading many studies to use indices or minimum population sizes as a metric of comparison^26, 28^.

The range of our density estimates (0.91–2.60 adult pigs/km^2^) is consistent with published estimates of wild pig density in the southeastern US (i.e. 1.07–2.74 pigs/km^2^)^29^, suggesting that severe overestimation or underestimation by field application of the tested density estimators did not occur. Our results suggest that the ecological process resulting in scales of movement can have a large effect on density estimates, as shown by higher rates of scaled bias across scale of movement ranges (Fig. 2); highlighting the importance of using effective post-hoc approaches to convert estimates of abundance to density^11^ or using techniques that implicitly consider spatial variation to estimate density. It should be noted, however, that the movement metrics observed from field data fell within a reasonable range for estimating density with fairly low bias from simulations (Fig. 2). In general, estimators performed exhibited greater bias and imprecision when scales of movement were low, which may partially be a result of fewer detections of individual animals at different detectors. Therefore, sampling design to maximize detections of individuals at multiple detectors, and thereby improve measurements of scale of movement will likely improve accuracy of density estimates. This might be implemented by placing detectors closer together for animals that generally exhibit lower movement rates and farther apart for species that tend to move larger distances. When capture rates were low as a result of extremely low baseline probabilities of detection or densities, all estimators suffered reduced performance, regardless of movement parameter values. Our simulations also suggest that MMDM, rather than HMMDM, should be used to convert estimates of abundance to density for greatest accuracy, similar to previous research^12, 15^ (Appendix S1). It should be restated, however, that there is no theoretical basis for use of MMDM as an appropriate buffer^15^, and that variation surrounding this estimate of an appropriate buffer size is not incorporated into the overall variation around the estimate of population density.

We found that LPEs generally estimated lower densities than other field techniques with relatively high precision; however, our simulations suggest that these estimates may be disputable. One assumption of LPEs is that marks are not lost or overlooked^5^, which was violated in the case of the biomarker LPE, and is likely to have affected the accuracy of density estimates (Appendix S2). It has previously been suggested that LPEs may be relatively unbiased when different methods of capture and recapture are implemented to reduce effects of individual heterogeneity^5^, which may explain the apparent accuracy of LPEs in our simulations. The relative accuracy of LPEs in simulations may also be partially accounted for by the fact that LPEs are known to perform well when home ranges are circular^11^, as implemented in our simulations. However, LPEs often had poor ability to correctly estimate error (Fig. 3) and do not accommodate model selection approaches, which may limit their utility in determining effects of specific covariates on density estimates^7^. LPEs were able to estimate densities even with low amounts of data, although the accuracy and precision of these estimates might be questionable. Overall, use of LPEs is likely most preferable when 1) a computationally simple method is necessary, 2) an assumption of circular home ranges is acceptable, 3) scale of movement and detection rates are fairly high, and 4) the researcher or manager is comfortable with some degree of inaccuracy and/or imprecision.

Camera SECR and trap SECR methods resulted in the highest density estimates and performed similarly under field conditions and in simulations. It is not unexpected that this would be the case, as corral trap data did not significantly change the models, but simply represented additional data that could be used to estimate scale of movement and detection parameters to better inform density estimates. Trap SECR models were generally more accurate and precise than camera SECR models as a result of this additional data. Spatially explicit models had the additional benefit of allowing incorporation of covariates to better account for underlying mechanisms that influenced the detection process (e.g. scale of movement), although these models also required a greater amount of data than other methods tested, and failed to run when insufficient data were available. This implies additional effort may be necessary to implement SECR methods in the field compared to the other tested techniques, particularly when movement rates are low. Similar to other studies, SECR models were relatively imprecise under field conditions (Fig. 1), likely due to their incorporation of spatial variation into the estimation process^15^. Despite this, our simulations suggest that SECR models will be relatively precise when density, scales of movement, and/or detection rates are high, criteria that were not fully met by field data. Accuracy and precision of SECR models could be additionally improved by better tailoring the sampling grid design to reduce mismatches between perceived and actual movements, as discussed in ref. 30. It should, however, be noted that our SECR models were based upon an assumption of stationary home range centroids as our camera trapping study period was less than two weeks. However, if we had a longer study period, transience of animals through the study area could affect our results, although SECR model estimates should be generally robust to transience^31^. Based upon our results, we recommend SECR approaches be employed when 1) recaptures at multiple spatial locations are likely, 2) fairly accurate and precise density estimates are required, and 3) mismatches between grid size and movement patterns of animals are unlikely or can be minimized.

Development of removal models suggests that they can generate robust estimates of abundance^8^, however they do not inherently consider space, necessitating estimation of the effective area sampled by this technique through external data sources (e.g. use of remote cameras, telemetry) to allow density estimation. As expected, the buffer used for conversion to density must be realistic and preferably based on site-specific observations in order for good estimates to be obtained. While the removal models were somewhat biased when density and scales of movement were low, they exhibited high accuracy when population density was large and capture rates were sufficiently high. As expected based upon simulations, this estimator performed poorly for the mixed study site, where capture rates were extremely low. This technique also had the lowest data requirements, needing only a simple count of animals removed during the study period and the effort required to remove them (here, trap nights). We believe that removal estimators will be most effectively employed when 1) population densities are fairly high and a reasonable capture rate can be attained^8^, 2) a simple method of data collection is preferred, 3) the target population is already being managed by culling, and 4) data on movements of animals in the study area can be gathered or inferred.

When choosing the most appropriate method to monitor populations, understanding the strengths and weaknesses of each technique is necessary. While we were able to individually identify animals of this species using photographs, the proportion of naturally marked and identifiable animals is likely to differ across regions and species. When unidentifiable individuals are present in the population, spatially explicit mark-resight methods that account for the proportion of unidentifiable individuals captured in photographs might offer a solution^3, 32^. Camera traps are already commonly used in many control programs for invasive and harvested species to assess presence and composition of populations prior to implementation of management strategies, suggesting camera-based methods could be an efficient technique for management applications.

A challenge with the biomarker-based method was that it was difficult to determine from camera-trap data whether individuals had consumed sufficient biomarker to be marked. This led to uncertainty in the number of marked animals within each study site, which could influence population estimates (Appendix S2). Using greater concentrations of biomarker, requiring less consumption by each individual to generate a mark, and/or a shorter marking period, as in^24^, may improve results. However, higher concentration of some biomarkers could reduce palatability of bait or make consumption unsafe for non-target species, requiring further modifications of bait matrix for success. We also found uncertainty in the recapture occasion of this technique, which may be due to variation in biomarker consumption among animals. Thus, as currently implemented, the biomarker technique likely needs further development to reduce observational error for effective implementation in density estimation.

Trapping is a commonly used technique to manage invasive and harvested species^33^, and use of trapping to estimate density, such as in the removal model, biomarker LPE, camera LPE, or trap SECR method we employed, is attractive as it may complement management programs^8^. To better estimate the area sampled by detectors and further refine density estimates from methods using trapping data, researchers might consider collecting external telemetry data to estimate the amount of time spent by individuals in a sampling area^11^. This will allow improved density estimation by techniques that do not explicitly consider movement and space, and may improve estimates of those that do.

Although we conducted this study over a relatively short period of time, the age structure of populations differed dramatically between study sites (Appendix S1). Estimates of density that include young could change greatly within a few months in species that exhibit birth pulses, or across space in species that breed year round, necessitating careful interpretation of results or increased planning to account for temporal and spatial variation in births. We also believe future studies of social species, such as wild pigs, should investigate the independence of adult animals within the same group to ensure independence of samples or assess the necessity of modification to density estimation techniques. In addition, improved information about reproductive parameters, such as the proportion of animals reproducing and average litter size, could be used to incorporate non-independent juveniles into estimates. To our knowledge, no study has extensively evaluated the degree to which wild pigs of the same social group are spatially independent (i.e., the amount of time that animals of the same group do not spend together and might be detected independently). Development of methods to incorporate spatial auto-correlation at the individual level might be valuable for future studies of this and other social species.

## Methods

### Study Species

Wild pigs (*Sus scrofa*) and wild boar, from which they are descended, are found on every continent except Antarctica^34^. This species is often harvested recreationally and lethally controlled in locations where it is invasive. There is well-recognized bias in capture probabilities of different demographic components of wild pigs through conventional trapping^33^ and it is likely that movement rates of this species differ between habitat types.

### Study Area

**Figure 4:**
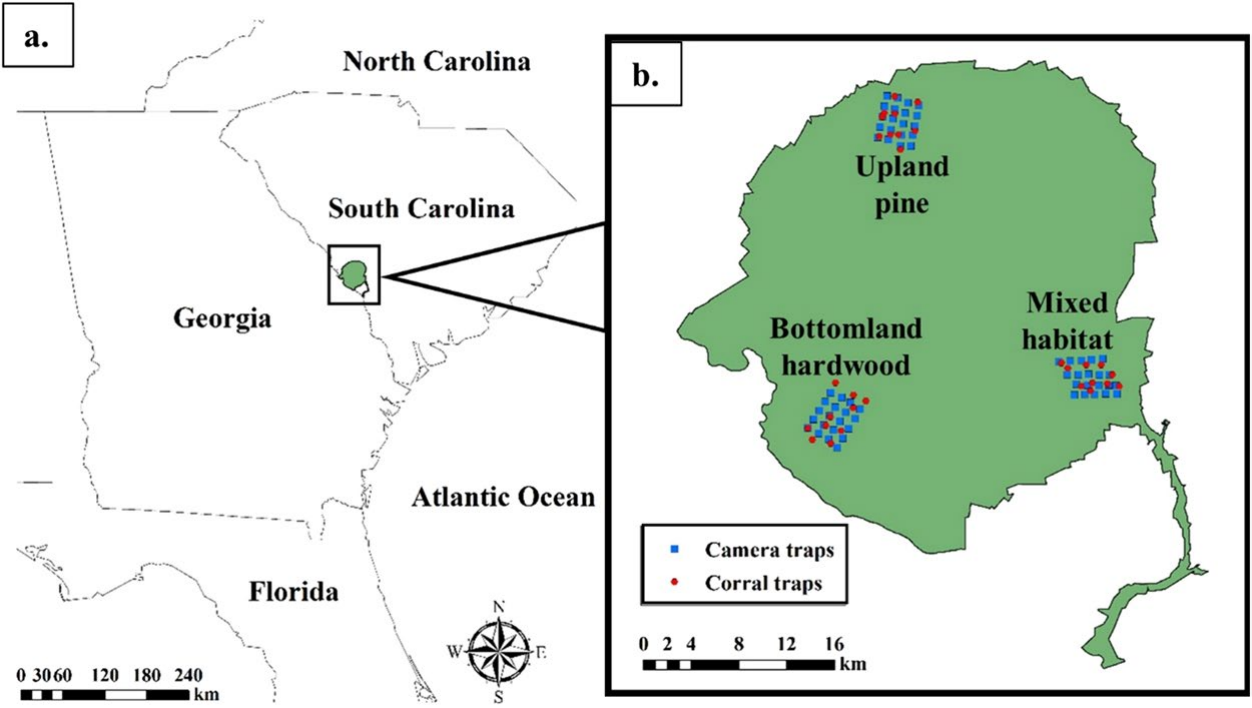
Location of the Savannah River Site (**a**) and distribution of detectors in the selected study sites (**b**), South Carolina, USA, 2015. Maps were created in ArcMap^49^.

We conducted this research at the Savannah River Site (SRS), a 78,000 ha United States Department of Energy (DOE) facility on the border of South Carolina and Georgia (33°20′N, 81°44 W; Fig. 4). Approximately 68% of habitat at the SRS consists of upland pine, while an additional 22% is comprised of swamp and riparian bottomland habitat (described in ref. 35). Additional areas, hereafter mixed habitat, are dominated by upland pine, but include riparian habitat. We selected a study site in each of these three broad habitat matrices (i.e. bottomland hardwood, upland pine, and mixed habitat) to test density estimators under varying field conditions. Populations of wild pigs on the SRS have grown recently as evidenced by increasing incidences of pig-vehicle collisions and numbers of individuals culled by U.S. Forest Service contractors^36^.

### Field Methods

All field methods were carried out in accordance with approved guidelines and research protocols (University of Georgia IACUC permit A2015 05–004-Y). Within each study site (bottomland, mixed, upland), we applied three common field techniques to gather data. These techniques were 1) individual identification of animals using camera traps and natural marks, 2) use of a biomarker bait to mark individuals for capture-recapture analysis, and 3) application of trapping and lethal removal. Camera trapping and biomarkers were simultaneously applied in each study site prior to live-trapping. Each of the combined field and analytical methods we evaluated was self-contained (i.e. did not require capture and marking of individuals prior to implementation or gathering of external data, such as telemetry). Table 1 provides an overview of how field data fed into the analytical methods tested.

**Figure 5:**
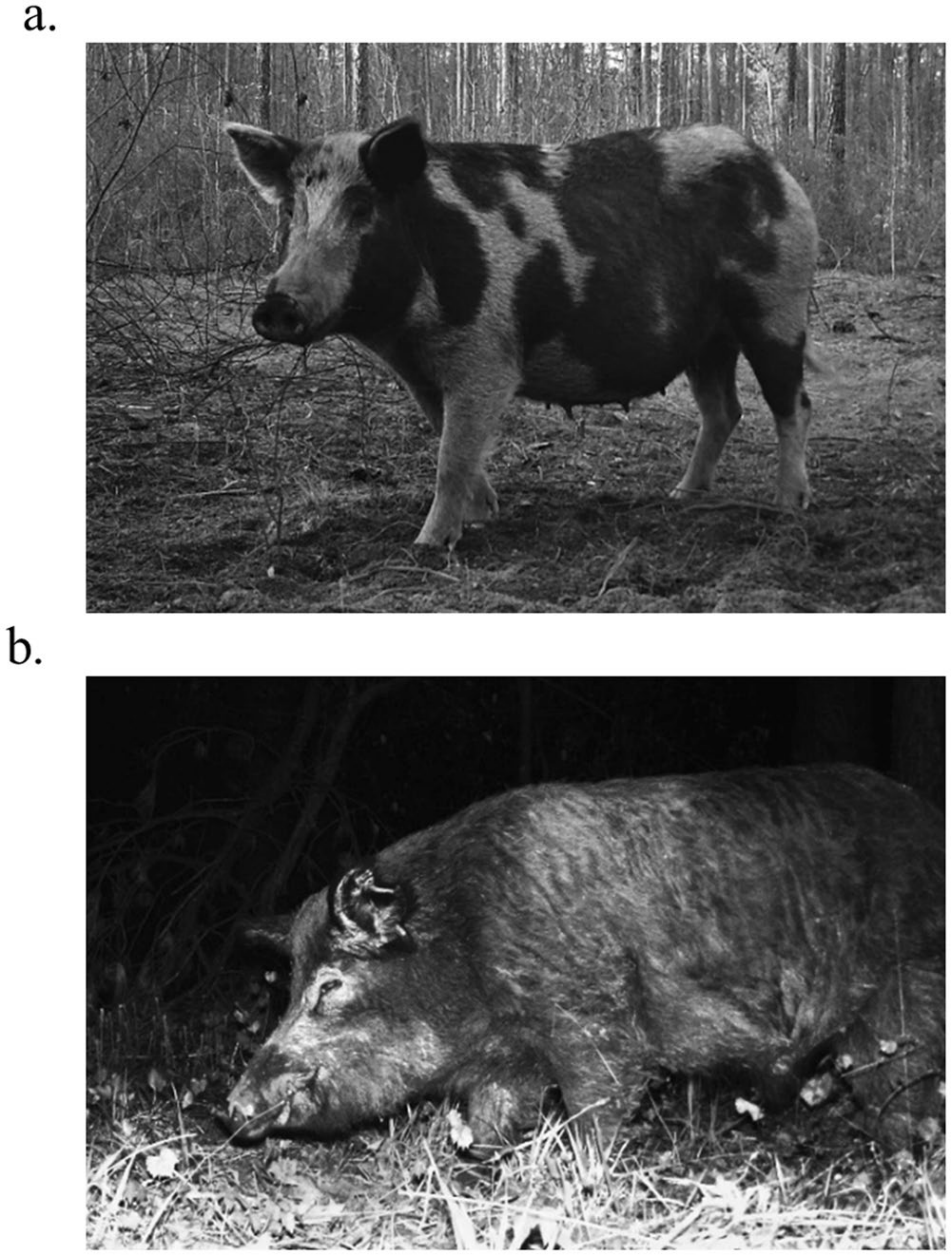
Example of a wild pig (*Sus scrofa*) individually identified by pelage patterns (**a**) and a wild pig identified by scars at a Rhodamine B treated bait pile (**b**), Savannah River Site, South Carolina, USA, 2015.

We established a 5 × 4 grid of white-flash trail cameras (Scoutguard SG565FV, HCO Outdoor Products, Norcross, USA; Fig. 4) in each study site. We placed cameras along transects 750 m apart (±7  m) in locations that would maximize the probability of animal detections based upon local habitat conditions or evidence of pig presence (e.g. rooting, scat, etc.). Cameras were set on motion triggers, with a 3-minute delay between trigger activation, and programed to take 3 pictures, 5 seconds apart, when triggered. We baited cameras with corn treated with Rhodamine B (RB), a biomarker that can be used for “batch-marking” individuals prior to removal efforts^37^ (described in Appendix S3 in Supporting Information). Camera traps were active for 12 days in the upland and mixed study sites, and 13 days in the bottomland study site. We identified individual animals using unique combinations of pelage, scars, and association with other individuals from camera photos (Fig. 5). To create individual capture histories, each 24-hour period a camera was active defined a capture occasion. Using camera trap data, we determined whether each individual pig was likely to be marked by RB through evaluation of the amount of time it spent consuming treated bait and its estimated weight (Appendix S3). We assessed accuracy of our classifications of animals as “marked” or “unmarked” based upon their consumption of RB by determining how many individuals that were judged marked were not marked based upon whisker analysis (i.e. false positives), and how many animals thought to be unmarked were marked according to whisker analysis (i.e. false negatives).

Following camera trapping, we created a grid of 1 km^2^ cells in each study site, and placed 10 corral traps (1 trap per grid cell) in areas with recent pig activity or in what was judged to be the best habitat if no fresh activity was found (Fig. 4). We pre-baited traps with whole corn for three days and live-trapping occurred for 14 days in each study site (i.e. 140 trap-nights per habitat type). To account for effort using traps, we recorded each occasion a trap was triggered without successfully catching a pig; these occasions were generally the result of a non-target species activating the trigger. Captured pigs were euthanized via cranial gunshot (University of Georgia IACUC permit A2015 05-004-Y). We collected 8–10 whiskers from each captured pig for use in analysis of RB consumption, and photographed each animal with a digital camera to allow identification of pigs that had previously visited camera traps. Whiskers collected from captured pigs were prepared for analysis according to the methods described in ref. 37. Further detail on the implementation of field protocols is available in Supporting Information (Appendix S3).

### Analytical Methods

Data sources for each analytical technique, method of implementation, and basic citations are in Table 1. We excluded individuals ≤20 kg from all analyses, as piglets travel with older individuals^28^, and would violate the independence assumption inherent in the estimation methods we used.

We assumed demographic and geographic closure existed among adult animals in each study site, as this study was conducted in a short time period (~1 month). Human harvest is frequently the largest source of adult mortality in wild pigs^38^, however, no hunting, vehicle deaths, or culling (outside our study design) occurred within ~2 km of the study sites during this project.

In the biomarker Lincoln-Petersen Estimator (LPE) and camera LPE, we calculated abundance of wild pigs using the Chapman correction for small sample size^6, 7^. Marked animals for the biomarker LPE were those that consumed a sufficient amount of RB (described in Appendix S3), whereas in the camera LPE, marked animals were those that were individually identified by camera trap photographs. The recapture occasion for both LPEs consisted of corral trapping and lethal removal of animals.

For the camera SECR analysis, we created and compared 10 *a priori* SECR models of wild pig density, available in Appendix S4. These models included potential factors affecting density (D), the scale parameter (sigma), describing how detection declines with distance between an animal’s home range center and a detector (i.e. camera), and the probability of detection (g0). These models assumed animals were distributed on the landscape following a homogenous Poisson point process, and that probability of detection was related to distance between an animal’s activity center and detectors through a half-normal curve^39^. We evaluated the level of support for each model using change in second order Akaike’s Information Criterion (∆AIC_C_) and AIC weight (AIC_*wi*_), measures of model likelihood^40^. We chose the results of the most supported model for comparison to the other population estimation techniques. If model selection uncertainty occurred, we used model averaging to estimate parameters.

For the trap SECR analysis, we used individual capture histories from the camera SECR method combined with live-trapping effort as additional potential capture occasions. We evaluated 10 *a priori* models of wild pig density using ΔAICc and AIC_*wi*_ (Appendix S4). Corral traps were considered a “proximity”-type detector to allow data analysis using R package *secr* ^41, 42^. This implies that multiple individuals could be captured by the same detector during a time period, which was facilitated by bait placement and using continuous-catch gates on many of the traps (Appendix S3). Similar to^18^, this assumption likely did not influence our estimates, as mean trap saturation, or the occasion specific proportion of occupied traps, was low (<0.03, Appendix S1). We also tested models that included a categorical effect of trap type (i.e. camera trap vs. corral trap) on detection probabilities.

For the removal method, we used a Bayesian hierarchical removal model, accounting for variation in capture effort, to estimate abundance in each study site^8^. The removal method was a standard removal model^43^ that jointly estimated capture rate and initial population size, and assumed changes in population size during the study were exclusively due to removals. Capture probability was dependent on the amount of effort (i.e. number of traps active in a given night). We implemented the model as in ref. 8.

### Converting abundance to density

To compare the techniques employed, we converted abundance estimates (i.e. biomarker LPE, camera LPE, removal) to density, as estimates of density are scalable across studies. We estimated the effective area sampled by each method as the area encompassed by the sampling grid buffered by the mean maximum distance moved^10^ (MMDM), as calculated using camera and corral trap capture data. We used the Delta method^44^ to calculate variances for the analytical techniques requiring a conversion from abundance to density (i.e. biomarker LPE, camera LPE, and removal). In addition, for the removal method we used a naïve buffer calculated from literature values for wild pig home range size^45^ to determine how this estimator performed without site-specific movement data.

### Simulation

We used simulations to evaluate the accuracy of each analytical method with varying densities, detection rates, and scale of movement parameters. We excluded the biomarker LPE in simulations due to observational process uncertainty (see Results).

We simulated a homogenous landscape and added a camera grid as implemented in the field component of this study (i.e. 20 cameras spaced 750 m apart in a 5 × 4 grid). We used a similar method to simulate trap locations (i.e. 10 traps in a spatially balanced design within the camera array). We then simulated spatial distributions of animal home range centroids using a partial Poisson clustering algorithm (R function *PCP.sim* ^46^) to account for the social dynamics of this species. We assumed that home range centroids were stationary throughout the study period (i.e. dispersal or transience were not evident)^31^.

Our observation process was based on a daily scale (i.e. any observation of an animal, regardless of the number of observations of that animal, within one day was treated as a single detection). There are many metrics that describe movement rates (e.g., step lengths, turning angles, maximum distance moved, hourly-, daily-, monthly- movement rates, etc.). While many of these movement metrics could influence the number of detections occurring within a single day, we wanted to focus on metrics that would relate to the probability of an animal being detected at any given detector within a day. We created an observational process in which the likelihood of an animal being available for detection depended upon the distance between its home range centroid and detectors (*d*). We assumed the probability of an animal being available for detection at a detector decreased with increased distance between that animal’s home range centroid and the focal detector, similar to distance sampling^47^ and a simplified movement process with a point of attractions^48^, which we implemented through a truncated Gaussian relationship (eq. 1). This method simplifies uncertainties associated with complex movement processes that might arise from a more explicit movement model (e.g., correlated random walks, Brownian Bridge) to the scale of movement metric (*σ*) which is important for this observational level.

1 $$p(availability)=\frac{1}{\sqrt{2\pi {\sigma }^{2}}}{e}^{-\frac{{d}^{2}}{2{\sigma }^{2}}}$$

We simulated the scale of movement metrics by varying the standard error (*σ*) of the truncated Gaussian distribution to affect the potential that an animal would be available for encounter with a detector. The maximum distances at which animals might encounter corral traps were simulated as being greater than those of camera traps, as there would be fewer detectors and, therefore, bait, present on the landscape during the trapping period, potentially causing animals to move greater distances. We modeled detection as a simple rate given the animal was available to be detected (e.g., the animal being within the proximity of the detector; *g*_0_, eq. 2). The detection probability was examined uniformly across a range of values from 0.1 to 0.9. The encounter rates were thus dependent on the movement process multiplied by the detection rates. This represents a scaled truncated Gaussian distribution (the combination of a maximum detection probability from a uniform range and a truncated Gaussian distribution, eq. 3). We restricted the total number of traps an animal could visit in a single night using a multinomial process based upon our observed empirical distribution of trap attendance and capture rates (eq. 4). In addition, we included a behavioral effect that increased the chances of an individual returning to a camera in subsequent nights once it was detected (i.e. “trap-happiness”), as supported by SECR model results (Appendix S4). In simulating corral trapping, animals could only be detected at one trap ever, and then were removed from the population.

2 $$p(detection|available)={g}_{0}$$

3 $$p(detection)={g}_{0}\,\ast \,\frac{1}{\sqrt{2\pi {\sigma }^{2}}}{e}^{-\frac{{d}^{2}}{2{\sigma }^{2}}}$$

4 $$\#\,traps|detected=multinomial(\#detected,[0.82,\,0.15,0.03])$$

We simulated all combinations of a range of scale of movement (0.1, 0.2, 0.4, 0.6, 0.8, 1.0, 1.2), density (0.25, 0.50, 0.75, 1.25, 2.00, 2.50, 3.75, 5.00, 6.25, 7.50, 10.00, 15.00), and detection probabilities (0.1, 0.2, 0.3, 0.5, 0.7, 0.8, 0.9), for a total of 588 combinations. We generated five sample datasets from each combination. We defined scale of movement (sigma) as the standard deviation of a Gaussian distribution, based upon the average radius of two week home range sizes from collared animals at the SRS (Smith *et al*., Savannah River Ecology Laboratory, unpublished data). The detection probability was modeled as being 75% lower for corral traps than camera traps based upon field data, meaning that given the distance between its home range centroid and the detector was the same, the probability of a pig being captured in a corral trap was ¼ of its probability of being captured at a camera. We compared analytical methods in terms of scaled bias, the deviation of the estimated density from the true density used to create the simulation, and scaled by the true density, and coefficients of variation of the density, a scaled measure of variability representing relative precision.

## Electronic supplementary material

Appendix S1-S5
