# Supplementary material for: Effects of scale of movement, detection probability, and true population density on common methods of estimating population density
Source: Sci Rep. 2017 Aug 25;7:9446. doi: 10.1038/s41598-017-09746-5 (PMC5573344; doi:10.1038/s41598-017-09746-5)
Supplement: Supplementary file 1 — Appendix S1-S5 [file 41598_2017_9746_MOESM1_ESM.pdf]

Appendix S1

Supplementary information

Effects of scale of movement, detection probability, and true population density on common methods of estimating population density

Dave Keiter[1*], Amy Davis[2], Olin E. Rhodes Jr.[3], Fred Cunningham[4], John Kilgo[5], Kim Pepin[2†], and Jim Beasley[1†].

[1]*University of Georgia, Savannah River Ecology Laboratory, D.B. Warnell School of Forestry and Natural Resources, PO Drawer E, Aiken, SC 29802, USA*

[2]*United States Department of Agriculture, Animal and Plant Health Inspection Service, Wildlife Services, National Wildlife Research Center, 4101 Laporte Avenue, Fort Collins, CO 80521, USA*

[3]*University of Georgia, Savannah River Ecology Laboratory, Odum School of Ecology, PO Drawer E, Aiken, SC 29802, USA*

[4]*United States Department of Agriculture, Animal and Plant Health Inspection Service, Wildlife Services, National Wildlife Research Center, Mississippi Field Station, PO Box 6099, Mississippi State, MS 39762, USA*

[5]*United States Department of Agriculture, Forest Service, Southern Research Station, PO Box 700, New Ellenton, SC 29809, USA*

[*]*Corresponding author: Dave Keiter, david.keiter@gmail.com*

[†]*These authors made equal contributions*

Additional discussion of results

1. Camera trapping

We were able to identify each wild pig that encountered a camera trap in this study for the purpose of creating capture histories, based upon size, pelage, scarring, and associated group members[1]. We did not observe evidence of animal wariness regarding camera traps, resulting in high rates of identification, although a small proportion of photographs contained an individual that was most likely marked, but unidentifiable due to distance or angle of the camera relative to the pig. Similar to [2], we excluded these individuals from analysis. We identified a total of 51 individual adult and subadult pigs and 25 piglets across the three study sites (Supplementary Table S1, Supplementary Fig. S1). Greater numbers of piglets were identified in the upland site than the bottomland or mixed sites. Piglets were photographed with associated adult or subadult wild pigs at almost every capture occasion.

The most supported camera SECR model included an effect of behavior and a linear time trend on probability of detection, and differing scale of movement rates between study sites ($AIC_{wi} = 0.91$; Supplementary Information Appendix S4). The most supported trap SECR model included these same effects and an additional effect of detector type (i.e. camera trap vs. corral trap) on probability of detection ($AIC_{wi} = 0.94$; Appendix S4). These results provide evidence of "trap-happiness" in wild pigs visiting baited camera traps, and evidence of differing capture probabilities between camera and corral traps.

2. Biomarker use

Of the identified adult and subadult wild pigs, hereafter adults, we estimated that 46 individuals were marked through consumption of Rhodamine B (RB) based upon camera trap imagery (Supplementary Fig. S1). Analysis of whisker samples showed that of the 31 adults captured in corral traps, 26 (83.87%) were judged to have consumed RB. Of the animals captured in corral traps, 2 pigs were thought to have been marked based upon camera observations and were not marked based upon whisker analysis, giving a false positive rate of 6.45%, while 4 pigs were thought to have not consumed sufficient RB to be marked, but were positive by whisker analysis, giving a false negative rate of 12.90%. We present a file of R code that can be used in a sensitivity analysis to assess the potential effects of observer error on density estimates (Appendix S2).

3. Live-trapping and euthanasia

In corral traps, 32 adult pigs and 27 piglets were captured across the three study sites (Supplementary Table S1). In total, 85 unique pigs were identified by camera traps, capture in corral traps, or both. Of the pigs captured in corral traps, the majority (84.74%) were identified on at least one camera trap prior to their capture in a corral trap. Mean trap saturation (occasion specific proportion of occupied traps) for the three study sites is presented in Supplementary Table S2.

Buffer estimation

Scale of movements as determined by camera and corral trap data were variable for pigs in all study sites, however, the highest mean maximum distance moved (MMDM) occurred in the bottomland site (897.37 m, SE = 151.09), while pigs in the upland study site (MMDM = 647.32 m, SE = 161.83) and mixed site (MMDM = 327.81 m, SE =

116.99) appeared to move shorter distances. For this reason, the effective area sampled that was used to convert abundance to density was greater for the bottomland site than the upland or mixed site. Supplementary Fig. S2 and Supplementary Fig. S3 present comparisons of the scaled bias in density estimates of the removal model using MMDM, HMMDM, and a naïve buffer size generated from literature values[3].

Simulations

Supplementary Fig. S4 below depicts the effects of scale of movement parameters, probabilities of detection, and densities on the coefficients of variation of estimated densities by the tested analytical techniques, when the values of coefficients of variation are unconstrained.

Supplementary Table S1. Total captures of wild pigs (*Sus scrofa*) by study site and method, Savannah River Site, South Carolina, USA, 2015.

Habitat Type	# of Adults and Subadults (Camera)	# Piglets (Camera)	#of Adults and Subadults (Corral)	# Piglets (Corral)	# Trapped, not photographed
Bottomland hardwood	24	7	18	9	5
Mixed habitat	13	0	5	1	2
Upland pine	14	18	9	17	2
Total	51	25	32	27	9

Supplementary Table S2. Study-site specific mean trap saturation and standard error, Savannah River Site, South Carolina, USA, 2015.

Study site	Mean trap saturation	Standard error
Bottomland hardwood	0.027	0.015
Mixed habitat	0.013	0.009
Upland pine	0.04	0.021
Total	0.027	0.015

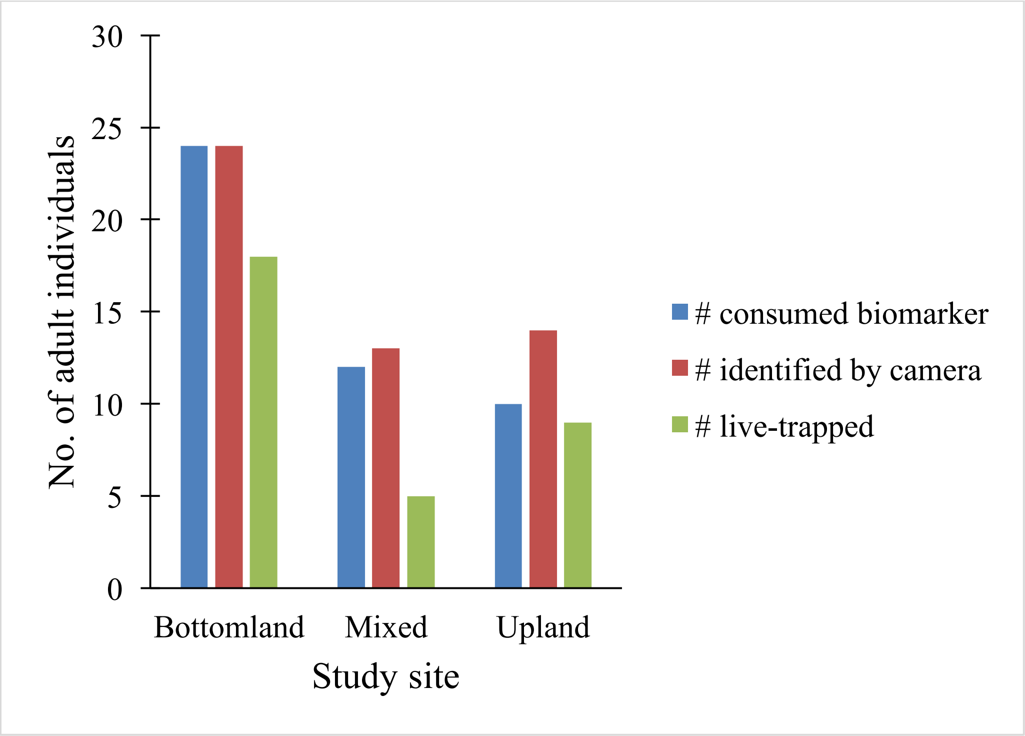

Supplementary Figure S1. Number of captured adult and subadult wild pigs (*Sus scrofa*) by field technique, Savannah River Site, South Carolina, USA, 2015.

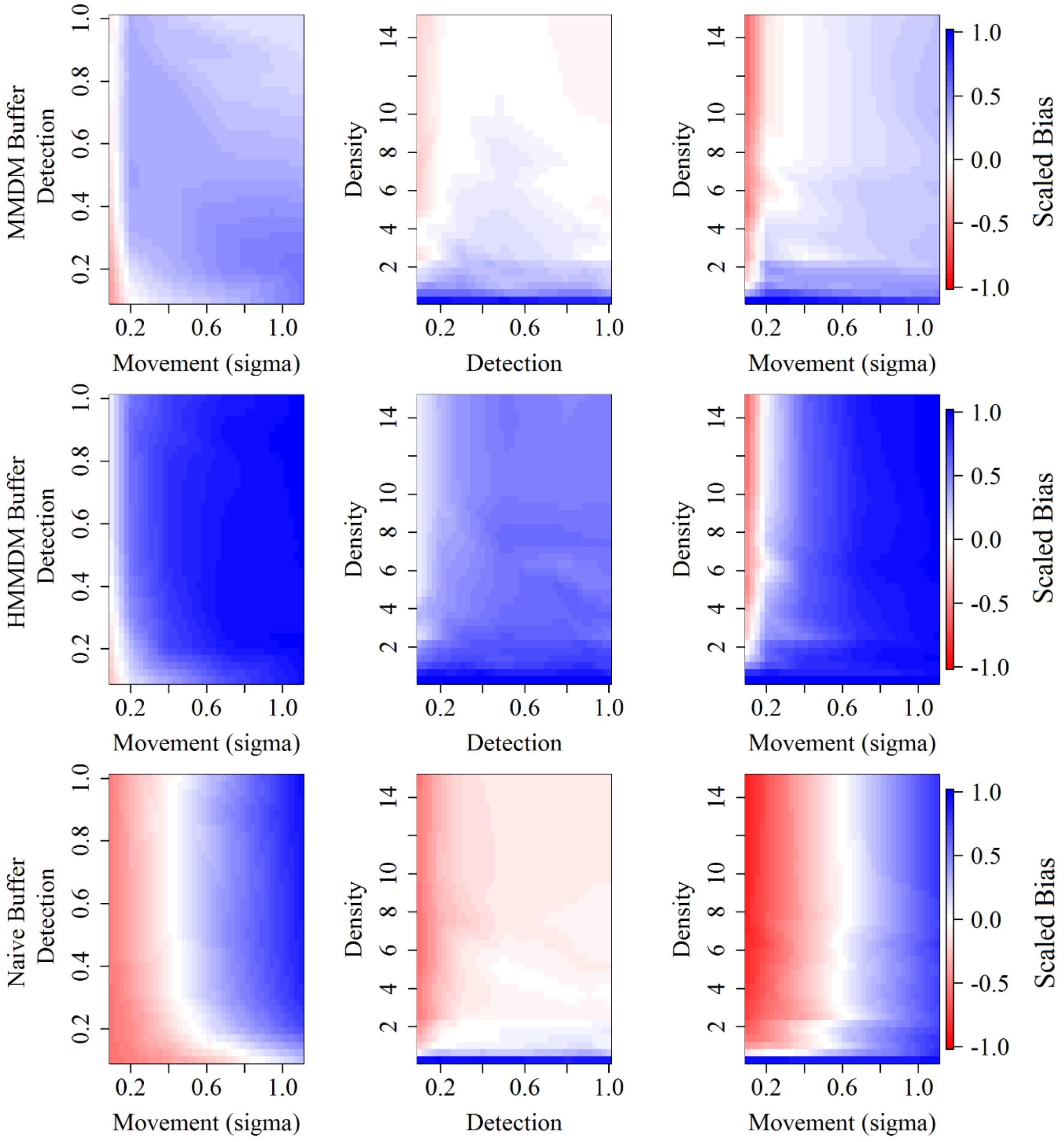

Supplementary Figure S2. Scaled biased for the removal model using a mean maximum distance moved (MMDM) buffer (top row), half mean maximum distance moved (HMMDM), and a naïve buffer size (bottom row).

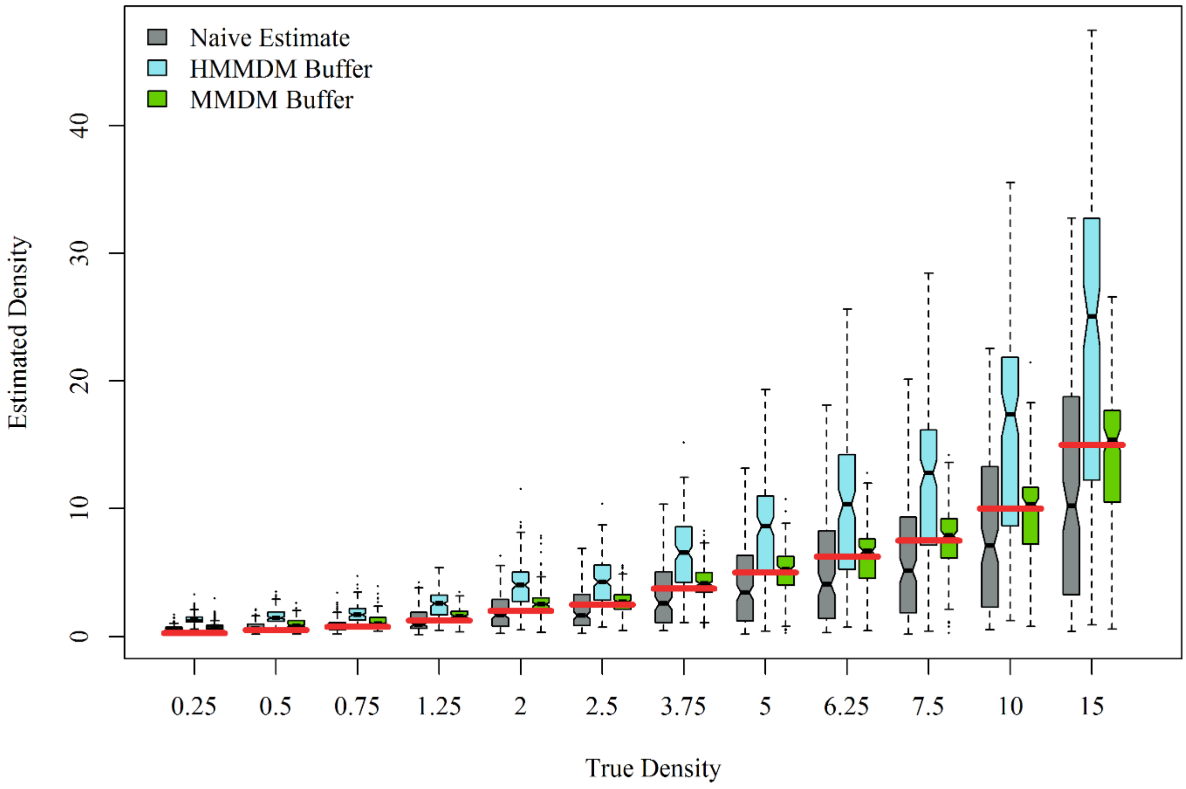

Supplementary Figure S3. Effects of buffer choice for the removal model. The red lines in the graph above depict the true density.

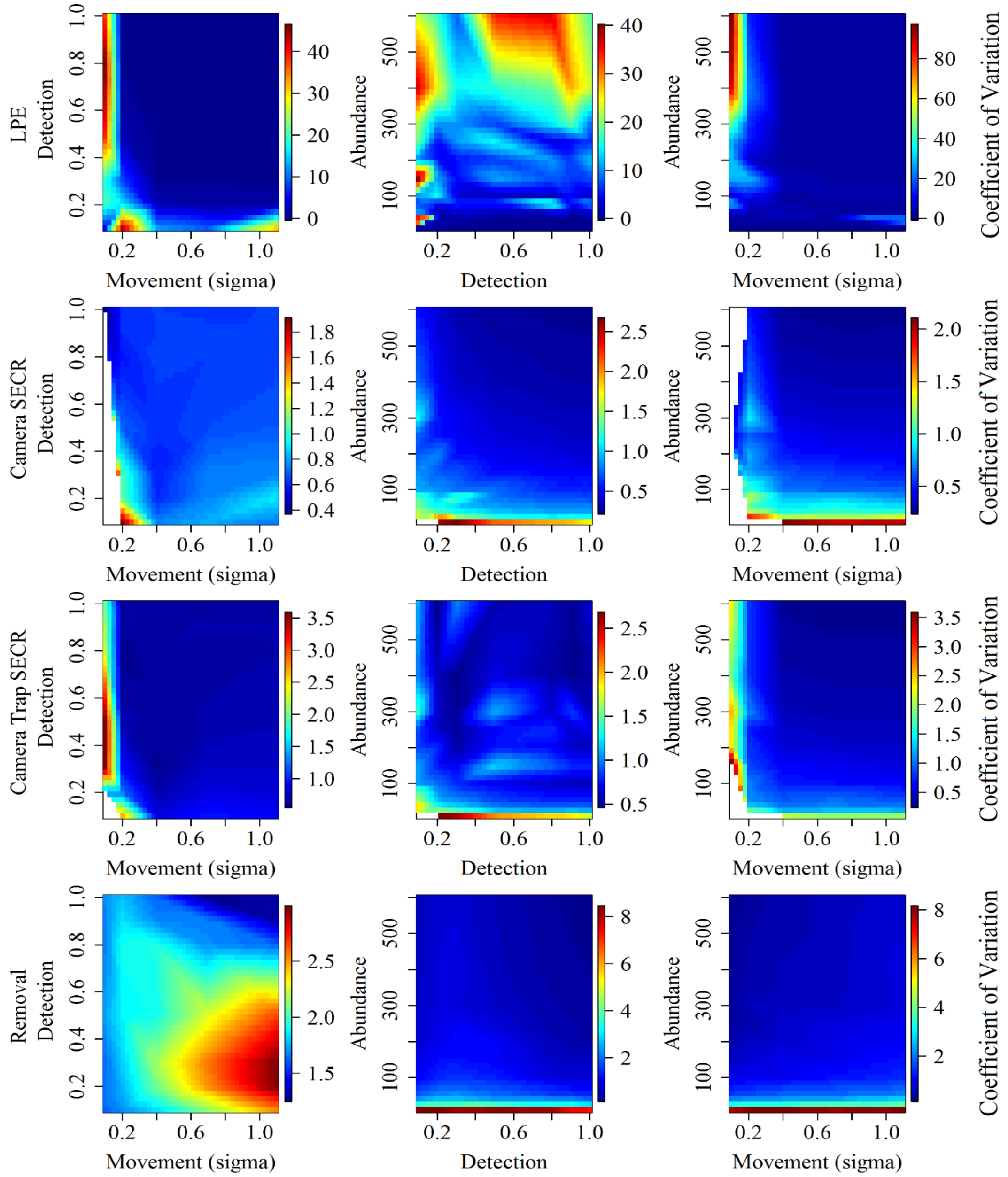

Supplementary Figure S4. Effects of scale of movement parameters, probabilities of detection, and densities on unconstrained coefficients of variation of estimated densities by the tested analytical techniques: camera LPE, camera SECR, trap SECR, and removal models from simulations. The scale of coefficient of variation values varies. Combinations of parameters at which models did not run are displayed in white.

Literature cited

1. Williams, B. L., Holtfreter, R. W., Ditchkoff, S. S. & Grand, J. B. Trap style influences wild pig behavior and trapping success. *Journal of Wildlife Management* **75,** 432-436 (2011).

2. Sweitzer, R. A., Van Vuren, D., Gardner, I. A., Boyce, W. M. & Waithman, J. D. Estimating sizes of wild pig populations in the north and central coast regions of California. *Journal of Wildlife Management* **64,** 531-543.

3. McClure, M. L. *et al*. Modeling and mapping the probability of occurrence of invasive wild pigs across the contiguous United States. *PLoS ONE* **10,** e0133771 (2015).

```
#################################################################
######
###
### Keiter et al. " Effects of scale of movement, detection probability, and true population
### density on common methods of estimating population density "
### Supporting Information 2
### Shiny app for sensitivity of Rhodamine B uptake by pigs
###
### Amy J Davis
###
#################################################################
######

library(shiny)
library(vioplot)

#### Create a LPE function

lpe.func<-function(m,c,r){
  N.hat=((m+1)*(c+1))/(r+1)-1
  N.hat
}

##### Shiny app code
ui <- fluidPage(
  h1("Sensitivity to violations of assumptions"),
  sidebarLayout(sidebarPanel(  sliderInput("PopSize",label="Abundance",10,1000,value =
200),
                      sliderInput("CapRate",label="Capture Rate",0,1,value = 0.3),
                      sliderInput("atetoomuch",label="False Positive Marks",0,1,value = 0.1),
                      sliderInput("atetoolittle",label="False Negative Marks",0,1,value = 0.1),
                      sliderInput("toopink",label="False Positive Recaptures",0,1,value = 0.1),
                      sliderInput("toolittlepink",label="False Negative Recaptures",0,1,value =
0.1),
                      helpText("True = estimates without bias"),
                      helpText("False Positive Marks = We say pigs ate enough bait but did not"),
                      helpText("False Negative Marks = We say pigs did not eat enough bait but
did"),
                      helpText("False Positive Recaptures = We say pigs are marked but are
not"),
                      helpText("False Negatives Recaptures = We say pigs are not marked but
are"),
                      helpText("All = All types of bias in one model")),
            mainPanel(plotOutput("DensityPlot",width="100%",height = 800,inline = FALSE)))
)
server <- function(input, output){
```

```
  output$DensityPlot<-renderPlot({
    par(mfrow=c(2,1),mar=c(4,5,1,1)+0.1)
    nhats=matrix(0,1000,6)
    for(i in 1:1000){
      ### Create the data frame

pigs=data.frame(ID=1:input$PopSize,TrueMark=rbinom(input$PopSize,1,input$CapRate),T
rueRecap=rbinom(input$PopSize,1,input$CapRate))
      ### Was the pig marked and recaptured?
      pigs$TrueMR=pigs$TrueMark*pigs$TrueRecap

      ### False positive marks (saying a pig ate enough bait but did not)

pigs$SayMarkedHigh=ifelse(pigs$TrueMark==1,1,rbinom(input$PopSize,1,input$atetoomu
ch))

      ### False negatives marks (saying a pig did not eat enough but it did)
      pigs$SayMarkedLow=ifelse(pigs$TrueMark==0,0,rbinom(input$PopSize,1,(1-
input$atetoolittle)))

      ### False positive recaptures (saying a pig is Rhodamine B positive but it is not)

pigs$SayMRHigh=ifelse(pigs$TrueRecap==0,0,ifelse(pigs$TrueMR==1,1,rbinom(input$Pop
Size,1,input$toopink)))

      ### False negative recaptures (saying a pig is Rhodamine B negative but it is positive)

pigs$SayMRLow=ifelse(pigs$TrueRecap==0,0,ifelse(pigs$TrueMR==0,0,rbinom(input$PopS
ize,1,(1-input$toolittlepink))))

      ### All Biases together
      pigs$SayMarks=ifelse(pigs$TrueMark==1,rbinom(input$PopSize,1,(1-
input$atetoolittle)),rbinom(input$PopSize,1,input$atetoomuch))

pigs$SayMR=ifelse(pigs$TrueRecap==0,0,(ifelse(pigs$TrueMR==0,rbinom(input$PopSize,1,
input$toopink),rbinom(input$PopSize,1,(1-input$toolittlepink)))))

      ### True values first
      nhats[i,1]=lpe.func(sum(pigs$TrueMark),sum(pigs$TrueRecap),sum(pigs$TrueMR))

      ### False positives marks

nhats[i,2]=lpe.func(sum(pigs$SayMarkedHigh),sum(pigs$TrueRecap),sum(pigs$TrueMR))

      ### False negatives marks
```

```
nhats[i,3]=lpe.func(sum(pigs$SayMarkedLow),sum(pigs$TrueRecap),sum(pigs$TrueMR))

     ### False positive recaptures
     nhats[i,4]=lpe.func(sum(pigs$TrueMark),sum(pigs$TrueRecap),sum(pigs$SayMRHigh))

     ### False negative recaptures
     nhats[i,5]=lpe.func(sum(pigs$TrueMark),sum(pigs$TrueRecap),sum(pigs$SayMRLow))

     ### All biases together
     nhats[i,6]=lpe.func(sum(pigs$SayMarks),sum(pigs$TrueRecap),sum(pigs$SayMR))
    }
    colMeans(nhats)
    xmax=max(nhats)
    xmin=min(nhats)
    ymax=max(apply(nhats,2,function(x)density(x)$y))
    par(family="serif",cex.lab=1.5,cex.axis=1.5)

plot(density(nhats[,1]),xlim=c(xmin,xmax),ylim=c(0,ymax),xlab="Abundance",main="",lwd
=2)
    for(i in 2:6){
     lines(density(nhats[,i]),col=i)
    }
    abline(v=input$PopSize)
    legend("topright",c("True","False Postive Marks","False Negative Marks","False Positive
Recaptures",
               "False Negative Recaptures","All"),lwd=4,col=1:6,bty="n",cex=1.5)

    ### plot 2
    library(vioplot)
    nbias=((nhats)-input$PopSize)/input$PopSize

    par(mar=c(7,5,1,1)+0.1)
    plot(1,1 ,xlim=c(0.5,6.5),ylim=c(range(nbias)),type="n",xlab="",ylab="", xaxt="n")
    for(i in 1:6){
     vioplot(nbias[,i],col=i,at=i,add=TRUE)
    }
    mtext(1,at=1:6,par("usr")[4]+2.5,text = c("True","False \n Postive \n Marks",
                           "False \n Negative \n Marks","False \n Positive \n Recaptures",
                           "False \n Negative \n Recaptures","All"),cex=1.5)
    abline(h=0,lty=2)
  })
}
shinyApp(ui = ui, server = server)
```

Appendix S3

Supplementary information

Effects of scale of movement, detection probability, and true population density on common methods of estimating population density

Dave Keiter[1*], Amy Davis[2], Olin E. Rhodes Jr.[3], Fred Cunningham[4], John Kilgo[5], Kim Pepin[2†], and Jim Beasley[1†].

[1]*University of Georgia, Savannah River Ecology Laboratory, D.B. Warnell School of Forestry and Natural Resources, PO Drawer E, Aiken, SC 29802, USA*

[2]*United States Department of Agriculture, Animal and Plant Health Inspection Service, Wildlife Services, National Wildlife Research Center, 4101 Laporte Avenue, Fort Collins, CO 80521, USA*

[3]*University of Georgia, Savannah River Ecology Laboratory, Odum School of Ecology, PO Drawer E, Aiken, SC 29802, USA*

[4]*United States Department of Agriculture, Animal and Plant Health Inspection Service, Wildlife Services, National Wildlife Research Center, Mississippi Field Station, PO Box 6099, Mississippi State, MS 39762, USA*

[5]*United States Department of Agriculture, Forest Service, Southern Research Station, PO Box 700, New Ellenton, SC 29809, USA*

[*]*Corresponding author: Dave Keiter, david.keiter@gmail.com*

[†]*These authors made equal contributions*

Additional description of field methods

1. Camera traps

In addition to baiting each camera trap with 11.4 kg of RB-treated bait, we used 11.4 kg of whole corn to create bait trails to increase initial detection probabilities of animals at camera stations[1]. We replaced depleted bait piles with 11.4 kg of whole corn, 5-6 days following deployment of cameras. Additionally, following the camera trapping period we removed any remaining bait, biomarker-treated or otherwise, in order to avoid influencing the success of trapping efforts. When identifying individual pigs (*Sus scrofa*) to create capture histories, we created a separate computer folder for each individual animal containing representative photographs of that animal from different angles to facilitate re-identification throughout the analysis period.

2. Biomarker use

Rhodamine B (RB) is a fluorescent dye that can be used as a biomarker for studies of wildlife. The use of RB was recently assessed in wild pigs with the conclusion that it would be appropriate to use as a measure of bait consumption[2]. RB consumption leaves a fluorescing mark in the whiskers of animals that have consumed it, allowing their identification through whisker analysis. We created the RB-treated bait used in this experiment by thoroughly mixing 5.0 g of RB, 0.5 L of water and 11.4 kg of whole corn in an 18.9 L plastic bucket at each bait site. For RB marking to appear in the whiskers of a pig, the pig must consume a minimum dosage of 5 mg RB/kg mass (J. C. Beasley, University of Georgia, unpublished data). Therefore, at the dosage we used, a 100 kg pig must have consumed 1.14 kg of treated corn to exhibit a mark. 100 kg is far above the average weight of a wild pig on the Savannah River Site (36.6 kg) [3], leading us to believe that pigs would likely consume sufficient amounts of RB-treated corn to be marked

(Supplementary Figure S5). Using camera imagery, we judged the amount of time that an animal spent at a bait pile consuming bait and its approximate weight to estimate whether it consumed a sufficient quantity of the treated bait to generate a mark. For example, if the pig weighed 50 kg, it must have consumed ~0.6 kg of bait to generate a mark; if we observed this 50 kg pig consuming bait for 15 minutes, it likely consumed more than 0.6 kg of bait, and therefore we considered it marked.

We conducted our whisker analysis according to previously established procedures[2]. We cleaned whisker samples separately using distilled water, dried them, and then mounted each sample on a microscope slide using Fluoromount (Sigma-Aldrich, Missouri, USA). Three observers then independently evaluated each slide using a BX61 fluorescent microscope (Olympus Life Science Solutions, Pennsylvania, USA) and the consensus score was used to determine presence or absence of RB in each pig.

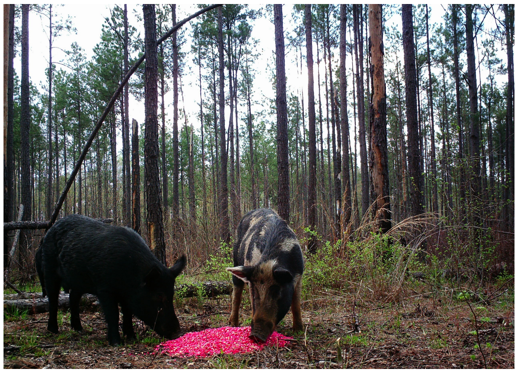

Supplementary Figure S5. Wild pigs (*Sus scrofa*) at a bait pile of Rhodamine B-treated whole corn, Savannah River Site, South Carolina, USA, 2015.

3. Live-trapping and euthanasia

We constructed all corral traps from metal paneling supported either by angle iron frames or t-posts with either a continuous-catch or guillotine-style gate (Supplementary Figure S6). Traps were triggered either by a root-stick or tripwire mechanism. We placed root-sticks and tripwires at a height greater than that estimated for piglets, in order to maximize capture success (as piglets frequently enter traps prior to adults and subadults). We baited traps in a manner to maximize capture success[4]. In addition to pre-baiting corral traps, we created bait trails of whole corn leading to traps in order to improve pig detection of trap locations.

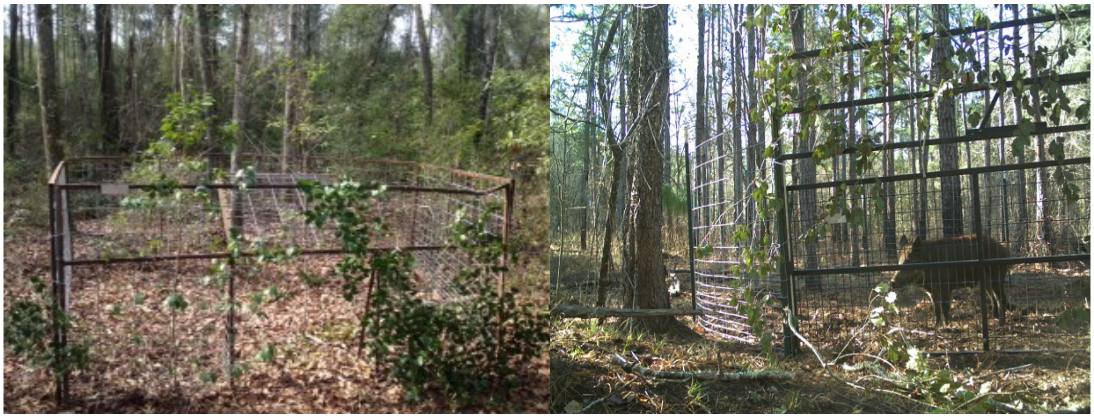

Supplementary Figure S6. Corral trap with continuous-catch or root gate (left) and wild pig (*Sus scrofa*) captured in a corral trap with a guillotine-style gate (right), Savannah River Site, South Carolina, USA, 2015.

References

1. Gerber, B. D., Karpanty S.M. & Kelly, M. J. Evaluating the potential biases in carnivore capture-recapture studies associated with the use of lure and varying density estimation techniques using photographic-sampling data of the Malagasy civet. *Population Ecology* **54,** 43-54 (2012).

2. Beasley, J. C., Webster, S. C., Rhodes Jr., O. E. & Cunningham, F. L. Evaluation of Rhodamine B as a biomarker for assessing bait acceptance in wild pigs. *Wildlife Society Bulletin* **39,** 188-192 (2015).

3. Mayer, J. J. & Johns, P. E. Characterization of wild pig-vehicle collisions in *Proceedings of the 12th Wildlife Damage Management Conference* (eds. Nolte, D. L., Arjo, W. M. & Stalman, D. H.).

4. West, B. C., Cooper, A. L. & Armstrong, J. B. Managing wild pigs: a technical guide. *Human-Wildlife Interactions Monograph* **1,** 1-54 (2009).

Appendix S4

Supplementary information

Effects of scale of movement, detection probability, and true population density on common methods of estimating population density

Dave Keiter[1*], Amy Davis[2], Olin E. Rhodes Jr.[3], Fred Cunningham[4], John Kilgo[5], Kim Pepin[2†], and Jim Beasley[1†].

[1]*University of Georgia, Savannah River Ecology Laboratory, D.B. Warnell School of Forestry and Natural Resources, PO Drawer E, Aiken, SC 29802, USA*

[2]*United States Department of Agriculture, Animal and Plant Health Inspection Service, Wildlife Services, National Wildlife Research Center, 4101 Laporte Avenue, Fort Collins, CO 80521, USA*

[3]*University of Georgia, Savannah River Ecology Laboratory, Odum School of Ecology, PO Drawer E, Aiken, SC 29802, USA*

[4]*United States Department of Agriculture, Animal and Plant Health Inspection Service, Wildlife Services, National Wildlife Research Center, Mississippi Field Station, PO Box 6099, Mississippi State, MS 39762, USA*

[5]*United States Department of Agriculture, Forest Service, Southern Research Station, PO Box 700, New Ellenton, SC 29809, USA*

[*]*Corresponding author: Dave Keiter, david.keiter@gmail.com*

[†]*These authors made equal contributions*

Tables presenting model comparison results for spatially explicit capture-recapture (SECR) models tested.

1. *Camera SECR*

Supplementary Table S3. Model selection results for *a priori* models of wild pig (*Sus scrofa*) density using spatially explicit analysis of camera trap data at the Savannah River Site, South Carolina, USA, 2015

Model	K[a]	AIC_C	ΔAIC_C	AIC_{wi}	-LL[b]
D[c] ~ 1<br>g0[d]~ b[e] + T[f]<br>sigma[g] ~ session[h]	7	1843.24	0	0.9186	-913.3177
D ~ session<br>g0 ~ b + T<br>sigma ~ session	9	1848.087	4.847	0.0814	-912.8486
D~session<br>g0~b<br>sigma~session	8	1858.31	15.07	0	-919.4407
D~1<br>g0~b+T<br>sigma~1	8	1873.453	30.213	0	-931.06

D~session<br>g0~b+T<br>sigma~1	5	1874.98	31.74	0	-929.1876
D~1<br>g0~b<br>sigma~1	7	1889.185	45.945	0	-940.1577
D~session<br>g0~b<br>sigma~1	4	1890.742	47.502	0	-938.4166
D~1<br>g0~T<br>sigma~1	6	2022.444	179.204	0	-1006.787
D~1<br>g0~1<br>sigma~1	4	2028.387	185.147	0	-1010.938
D~session<br>g0~1<br>sigma~1	3	2029.311	186.071	0	-1008.9889

D~session<br><br>g0~b<br><br>sigma~session	5	1843.24	0	0.9186	-913.3177

[a] = Number of parameters

[b] = Negative log-likelihood

[c] = Density

[d] = Probability of Detection

[e]= Effect of behavior

[f] = Linear time trend in detection

[g] = Distance between activity center and a detector

[h] = Study site

2. Trap SECR

Supplementary Table S4. Model selection results for *a priori* models of wild pig (*Sus scrofa*) density using spatially explicit analysis of camera trap and corral trap data at the Savannah River Site, South Carolina, USA, 2015.

Model	K[a]	AIC_C	ΔAIC_C	AIC_{wi}	-LL[b]
D[c] ~ 1<br>g0[d]~ b[e] + T[f] + Camera[g]<br>sigma[h] ~ session[i]	8	2244.67	0	0.9408	-1112.835
D~session<br>g0~b+T+Camera<br>sigma~session	10	2250.2	5.53	0.0592	-1112.708
D~1<br>g0~b+T+Camera<br>sigma~1	6	2267.739	23.069	0	-1127.03
D~session<br>g0~b+T+Camera<br>sigma~1	8	2268.641	23.971	0	-1124.821

D~1<br><br>g0~b+Camera<br><br>sigma~1	5	2290.187	45.517	0	-1139.505
D~session<br><br>g0~b+Camera<br><br>sigma~1	7	2291.323	46.653	0	-1137.518
D~1<br><br>g0~T+Camera<br><br>sigma~1	5	2455.39	210.72	0	-1222.107
D~1<br><br>g0~Camera<br><br>sigma~1	4	2456.333	211.663	0	-1223.782
D~session<br><br>g0~Camera<br><br>sigma~1	6	2457.534	212.864	0	-1221.927
D~1<br><br>g0~1<br><br>sigma~1	3	2528.03	283.36	0	-1260.789

[a] = Number of parameters

[b] = Negative log-likelihood

[c] = Density

[d] = Probability of Detection

[e]= Effect of behavior

[f] = Linear time trend in detection

[g] = Detector type (i.e. camera trap or corral trap)

[h] = Distance between activity center and a detector

[i] = Session is the term used for study site within our models

```
###############################################################################
#######
###
###  Appendix S5: R Code to simulate detections for density estimation comparisons – online
support
###     material for “Effects of scale of movement, detection probability, and true population
density on
###     common methods of estimating population density”
###
```

###David A. Keiter[1], Amy J. Davis[2], Olin E. Rhodes Jr.[3], Fred L. Cunningham[4], John C. Kilgo[5], Kim M. Pepin[2*], and James C. Beasley[1*]

###[1] University of Georgia, Savannah River Ecology Laboratory, D.B. Warnell School of Forestry and ###Natural Resources, PO Drawer E, Aiken, SC 29802, USA

###[2] United States Department of Agriculture, Animal and Plant Health Inspection Service, Wildlife ###Services, National Wildlife Research Center, 4101 Laporte Avenue, Fort Collins, CO 80521, USA

###[3] University of Georgia, Savannah River Ecology Laboratory, Odum School of Ecology, PO ###Drawer E, Aiken, SC 29802, USA

###[4] United States Department of Agriculture, Animal and Plant Health Inspection Service, Wildlife ###Services, National Wildlife Research Center, Mississippi Field Station, PO Box 6099, Mississippi ###State, MS 39762, USA

###[5] United States Department of Agriculture, Forest Service, Southern Research Station, PO Box 700, ###New Ellenton, SC 29809, USA

```
###Code by A.J. Davis
###############################################################################
#######

###
### Steps to follow
###  1) Start with the study area, camera grid, and trapping grid as specified in files
###  2) create a for loop to sample from each parameter (Density, Detection, and Distance)
###  3) For each sample generate 5 data sets under those conditions
###
### Load necessary libraries and source code
###
library(GISTools)
library(fields)
library(sp)
library(rgeos)
library(adehabitatHR)
library(rgdal)
library(secr)
library(gplots)
library(splancs)
```

```
library(lhs)
library(plyr)


###############################################################################
######
###
### 1) Get the study area and trap data
###
###############################################################################
######

#### Create the sampling area: a square polygon, 6.32 km x 6.32km (=40km^2)
spoly<-as.points(c(0,6.32,6.32,0),c(0,0,6.32,6.32)) #creates polygon object points
mypoly = SpatialPolygons(list(Polygons(list(Polygon(spoly)), "x")))  #creates spatial object of
the polygon

### Load the camera locations
camlocs=read.csv("camlocs.csv") #reads in previously created .csv file of camera locations
names(camlocs)<-c("CamID","x","y") #labels columns with ‘CamID’, ‘x’, and ‘y’
plot((camlocs)[,c("x","y")],col="red",pch=17,cex=2,
xlim=c(0,6.32),ylim=c(0,6.32),xlab="Km",ylab="Km") #plots uploaded camera locations

### Load the trap locations
traplocs=read.csv("traplocs.csv") #reads in previously created .csv file of trap locations
points(traplocs[,c("x1","x2")],col="green",pch=18,cex=3) #adds points representing trap
locations to plot

###################################
### Code to put a polygon around points
### then to put a buffer around the polygon
###
spdf=SpatialPointsDataFrame(coords = traplocs[,c("x1","x2")],data = data.frame(1:10)) #creates
spatial #points data frame of the coordinates of trap locations
sppoly=gConvexHull(spdf,byid = FALSE) #creates a convex hull polygon around coordinates
spbuff=gBuffer(spgeom = sppoly,byid=FALSE,width=1.123) #buffers convex hull polygon by
specified      #width
trapbuff=gArea(spbuff) #calculates area of the buffered polygon
plot(spbuff, add=TRUE) #plots the buffered polygon

### Creates a function to plot the traps and associated buffer
plotpigtrapsarea<-function(){
  spoly<-as.points(c(0,6.32,6.32,0),c(0,0,6.32,6.32)) # creates polygon object
  mypoly = SpatialPolygons(list(Polygons(list(Polygon(spoly)), "x")))  # spatial object of the
polygon

  ### Load the camera locations
```

```
  camlocs=read.csv("camlocs.csv") #reads in previously created .csv file of camera locations
  names(camlocs)<-c("CamID","x","y") #labels columns with 'CamID', 'x', and 'y'
  plot((camlocs)[,c("x","y")],col="red",pch=17,cex=2,
xlim=c(0,6.32),ylim=c(0,6.32),xlab="Km",ylab="Km")
  #plots uploaded camera locations

  ### Load the trap locations
  traplocs=read.csv("traplocs.csv") #reads in previously created .csv file of trap locations
  points(traplocs[,c("x1","x2")],col="green",pch=18,cex=3) #adds points representing trap
locations

  ####################################
  ### Code to put a polygon around points
  ### then to put a buffer around the polygon
  ###
  spdf=SpatialPointsDataFrame(coords = traplocs[,c("x1","x2")],data = data.frame(1:10)) #creates
spatial #points data frame of the coordinates of trap locations

  sppoly=gConvexHull(spdf,byid = FALSE) #creates a convex hull polygon around coordinates
  spbuff=gBuffer(spgeom = sppoly,byid=FALSE,width=1.123) #buffers convex hull polygon by
specified     #width
  trapbuff=gArea(spbuff) #calculates area of the buffered polygon
  ##plot(spbuff, add=TRUE) #plots the buffered polygon

} #closes function


################################################################################
######
###
### 2) Create data to sample from:
###
################################################################################
######
#### Combinatorics
### Abundance in an area that is 40km2
Nset=c(30,80,200,600) #creates a vector of the listed numbers
Nvec=rep(Nset,each=9) #repeats the vector a set number of times

### Sigma
sset=c(0.1,0.4,0.7) #creates a vector of the listed numbers
sigmavec=rep(rep(sset,by=4),each=3) #repeats the vector a set number of times

### Probability of detection
pset=c(0.2,0.5,0.8) #creates a vector of the listed numbers
phatvec=rep(pset,12) #repeats the vector a set number of times
```

```
###
### This is the set of data to sample
###
Value.set<-cbind(Nvec, sigmavec,phatvec) #binds all vectors together to create dataset of values
of N, #sigma, and phat


################################################################################
#
####
#### 3) Generate data to simulate 5 pig data sets for each sample
####
################################################################################
#

par(mfrow=c(1,1),mar=c(4,5,1,1)+0.1) #code to combine plots

### Set list of arrays for pig data
allpiggies=list() #creates a list object

#For-loop for running through values for sensitivity analysis
for(l in 1:dim(Value.set)[1]){
  N<-as.numeric(round(Value.set[l,1],0)) #alternates through values of N
  sig<-Value.set[l,2] #alternates through values of sigma
  detect<-Value.set[l,3] #alternates through values of phat

### Set a blank results array
  pigdata=array(0,c(N,2,5))


  ################################################################################
  ####
  #### Start of the loop to simulate 5 pig data sets and fit the removal model to them
  ####
  ################################################################################

  set.seed(13) #sets seed for generation of values
  #For-loop to run each analysis 5 times on the individual capture histories
  for(k in 1:5){

    #### Running a for loop to ensure the total population of pigs is 20, representing a density of
2.5 pigs/km2
    for(i in 1:100000){
      if(N>349){
        mypigs=pcp.sim(3.1,4,0.02,region.poly=spoly)
        dim(mypigs)[1]
```

```
    }else if(N>222&N<350){
      mypigs=pcp.sim(2.1,3,0.02,region.poly=spoly)
      dim(mypigs)[1]

    }else if(N>99&N<223){
      mypigs=pcp.sim(1.2,3,0.02,region.poly=spoly)
      dim(mypigs)[1]

    }else{
      mypigs=pcp.sim(.6,3,0.02,region.poly=spoly)
    }
    if(dim(mypigs)[1]==N){break}
  }
  dim(mypigs)[1]
  pigdata[,,k]=mypigs

  piggies=SpatialPointsDataFrame(coords = mypigs,data = data.frame(1:N))
 }
 allpiggies[[l]]=pigdata

}



#For-loop for running through values for sensitivity analysis
for(l in 1:dim(Value.set)[1]){
 cat("sample ",l," \n")
 N<-as.numeric(round(Value.set[l,1],0))
 sig<-Value.set[l,2]
 detect<-Value.set[l,3]


 ################################################################################
 ####
 #### Start of the loop to simulate 5 pig data sets and fit the removal model to them
 ####
 ################################################################################
 ### Set result matrix for removal data
 remdat=matrix(0,5,14)

 #For-loop to run each analysis 5 times on the individual capture histories
 for(k in 1:5){
  cat(k," ");flush.console()

  #### Running a for loop to ensure the total population of pigs is 20, representing a density of
2.5 # pigs/km2
  mypigs=allpiggies[[l]][,,k]
```

```
###
### ASSUME: availability is related to distance between homerange centroid and trap by a
half normal
### function
###

### Get data frame data
mypigsdat=data.frame(ID=1:N,mypigs)
mytraps=data.frame(traplocs) #creates data frame of trap locations
mycams=data.frame(Detect=1:20,camlocs[,c("x","y")]) #creates data frame of camera
locations

### Create distance matrices
dist.trap.pig=rdist(mypigsdat[,c("X1","X2")],mytraps[,c("x1","x2")]) #measure distance
between pig #activity center and trap locations
dist.cam.pig=(rdist(mypigsdat[,c("X1","X2")],(mycams)[,c("x","y")])) #measure distance
between pig #activity center and camera locations


################################################################################
#######
################################################################################
#######
###
### Create the camera trap capture histories
###
################################################################################
#######
################################################################################
#######

### Create an empty array for filling the camera x pig x time encounter indicator (1=yes, 0=no)
camcaps=array(0,dim = c(N,20,14))


### Standard deviation for camera radius of attraction
sdval<-sig #sets the standard deviation for camera radius of attraction to the value of sigma

### Standard deviation for trap radius of attraction
sdvalt=sig #sets the standard deviation for trap radius of attraction to the value of sigma
### Start out by generating whether a pig will be detected at all based the probability of being
detected by at least one trap
probs1=  exp(-1/(2*sdval^2)*dist.cam.pig^2)
pigprobs=(1-apply(probs1,1,function(x)prod((1-(x)))))*detect
```

```
### Distribution for number of traps if detected
trapsperpig=rmultinom(1,N,c(0.82,0.15,0.03))
ntraps=c(rep(1,trapsperpig[1,1]),rep(2,trapsperpig[2,1]),rep(3,trapsperpig[3,1]))

### Generate the number of traps that each pig was detected at
ntrappig=ifelse(runif(N)>pigprobs,0,1)*ntraps[sample(N)]


### Determine which traps the pigs were detected at for first trap night
for(i in 1:N){ #creates for loop to determine which traps pigs were detected at on the first trap night
  if(sum(exp(-1/(2*sdval^2)*dist.cam.pig[i,]^2))>0){
    camcaps[i,,1]=ifelse(rmultinom(1,ntrappig[i],exp(-1/(2*sdval^2)*dist.cam.pig[i,]^2))>0,1,0)
  }
}

### Create a for loop to create the encounter occasions for each of the subsequent time periods for #cameras
for(j in 2:14){
  pigfoundtrap=camcaps[,,j-1]

  ### Generate the number of traps that each pig was detected at
  ntrappig=ifelse(runif(N)>pigprobs,0,1)*ntraps[sample(N)]
  for(i in 1:N){

    camcaps[i,,j]=ifelse(rmultinom(1,ntrappig[i],exp(-1/(2*sdval^2)*dist.cam.pig[i,]^2))>0,1,0)

  }
  if(sum(pigfoundtrap)>0){
    ind1<-cbind(which(pigfoundtrap==1, arr.ind=TRUE),j)
    again=ifelse(runif(dim(ind1)[1])>0.5,0,1)
    camcaps[ind1]<-again
  }
}

### Create capture input file for camera data
pigcam=data.frame(Session=1,ID=0,Occasion=0,Detector=0)

for(i in 1:N){
  detects=which(camcaps[i,,]==1,arr.ind = TRUE)
  if(dim(detects)[1]>0){
    newpig=data.frame(Session=1,ID=i,Occasion=detects[,2],Detector=detects[,1])
    pigcam=rbind(pigcam,newpig)
  }
}
pigcam=pigcam[-1,]
```

```
###############################################################################
#######
###############################################################################
#######
  ###
  ### Create the trap capture histories
  ###
###############################################################################
#######
###############################################################################
#######

  ### Create an empty array for filling the camera x pig x time encounter indicator (1=yes, 0=no)
  trapcaps=array(0,dim = c(N,10,14))


  ### Start out by generating whether a pig will be detected at all based the probability of being
detected by at least one trap
  probst1=  exp(-1/(2*sdvalt^2)*dist.trap.pig^2)
  pigprobst=(1-apply(probst1,1,function(x)prod((1-(x)))))*(detect*0.25)

  ### Create a for loop to create the encounter occasions for each of the subsequent time periods
for cameras
  for(j in 1:14){
   ### Has the pig been killed?
   pigdead=apply(trapcaps,1,max) #determines if the pig was killed and is therefore unavailable
to be #trapped again

   ### Determine if the pig was trapped
   ntrappig=rbinom(N,1,pigprobst)
   for(i in 1:N){
    if(sum(exp(-1/(2*sdvalt^2)*dist.trap.pig[i,]^2))>0){
      trapcaps[i,,j]=ifelse(rmultinom(1,ntrappig[i]*(1-pigdead[i]),exp(-
1/(2*sdvalt^2)*dist.trap.pig[i,]^2))>0,1,0)
    }else {next}
   }
  }

  ### Create capture input file for trap data for SECR
  pigcamT=data.frame(Session=1,ID=0,Occasion=0,Detector=0)

  for(i in 1:N){
   detects=which(trapcaps[i,,]==1,arr.ind = TRUE)
   if(dim(detects)[1]>0){
```

```
      newpig=data.frame(Session=1,ID=i,Occasion=detects[,2],Detector=detects[,1])
      pigcamT=rbind(pigcamT,newpig)
    }
   }

   pigcamT=pigcamT[-1,]
   remdat[k,]=apply(trapcaps,3,sum)
   ###
   ### Format data for analyses
   ###
   pigcamT$Occasion=-(pigcamT$Occasion+14) #properly formats trap occasions to fit in with
camera data
   pigcamT$Detector=pigcamT$Detector+20 #formats detector number correctly for traps to fit
with camera data
   pigcamtrap=rbind(pigcam,pigcamT) #combines capture histories from cameras and capture
histories from traps
   camloc.out<-camlocs #creates new object from which coordinates can be extracted
   cam.coords<-camloc.out[c("x","y")]*1000 #extracts coordinates of camera traps

   traploc.out<-traplocs #creates new object from which trap coordinates can be extracted
   trap.coords<-traploc.out[c("x1","x2")] #extracts coordinates of corral traps
   trap.coords$x1<-trap.coords$x1*1000 #converts from kilometers to meters
   trap.coords$x2<-trap.coords$x2*1000 #converts from kilometers to meters
   trap.coords<-rename(trap.coords, c("x1"="x","x2"="y")) #renames the coordinates to allow
binding between camera and trap data

   camtrap.coords<-rbind(cam.coords,trap.coords) #combines trap and camera coordinates
  }
}
```
